# CX3CR1 deficiency promotes muscle repair and regeneration by enhancing macrophage ApoE production

**DOI:** 10.1038/ncomms9972

**Published:** 2015-12-03

**Authors:** Ludovic Arnold, Hélène Perrin, Camille Baudesson de Chanville, Marielle Saclier, Patricia Hermand, Lucie Poupel, Elodie Guyon, Fabrice Licata, Wassila Carpentier, José Vilar, Rémi Mounier, Bénédicte Chazaud, Nora Benhabiles, Alexandre Boissonnas, Béhazine Combadiere, Christophe Combadiere

**Affiliations:** 1 Sorbonne Universités, UPMC Univ Paris 06, Inserm, U1135, CNRS, ERL 8255, Centre d'Immunologie et des Maladies Infectieuses (CIMI-Paris), 91 Boulevard de l'Hôpital, F-75013 Paris, France; 2 Inserm, U1016, Institut Cochin, 22 Rue Méchain, F-75014 Paris, France; 3 CNRS, UMR8104, 22 Rue Méchain, F-75014 Paris, France; 4 University of Paris Descartes, Sorbonne Paris Cite, F-75006 Paris, France; 5 Sorbonne Universités, UPMC Univ Paris 06, Plateforme Post-Génomique de la Pitié-Salpêtrière (P3S), UMS2 Omique, INSERM US029, 91 Boulevard de l'Hôpital, F-75013 Paris, France; 6 Paris Centre de Recherche Cardiovasculaire (PARCC) – HEGP, 56 Rue Leblanc, F-75015 Paris, France; 7 CEA, List institute CEA Saclay, Digitéo Labs, PC192, F-91191 Gif-sur-Yvette, Cedex, France

## Abstract

Muscle injury triggers inflammation in which infiltrating mononuclear phagocytes are crucial for tissue regeneration. The interaction of the CCL2/CCR2 and CX3CL1/CX3CR1 chemokine axis that guides phagocyte infiltration is incompletely understood. Here, we show that CX3CR1 deficiency promotes muscle repair and rescues *Ccl2*^−/−^ mice from impaired muscle regeneration as a result of altered macrophage function, not infiltration. Transcriptomic analysis of muscle mononuclear phagocytes reveals that Apolipoprotein E (ApoE) is upregulated in mice with efficient regeneration. ApoE treatment enhances phagocytosis by mononuclear phagocytes *in vitro*, and restores phagocytic activity and muscle regeneration in *Ccl2*^−/−^ mice. Because CX3CR1 deficiency may compensate for defective CCL2-dependant monocyte recruitment by modulating ApoE-dependent macrophage phagocytic activity, targeting CX3CR1 expressed by macrophages might be a powerful therapeutic approach to improve muscle regeneration.

## Abstract (web summary)

Chemokine-driven infiltration of inflammatory macrophages is central to the muscle regenerative response to injury. Here the authors show that the function of infiltrating macrophages is also important as notexin-induced muscle injury in mice is rescued by CX3CR1 knockout owing to enhanced ApoE production.

## Graphical abstract

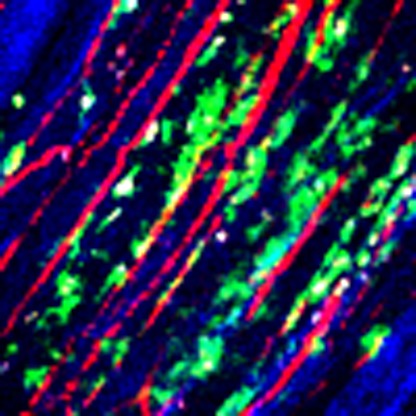

Monocytes are key players during trauma and inflammation, whereas tissue-resident macrophages have an important role during tissue homeostasis and the resolution of inflammation. However, it is not well established how monocytes and macrophages contribute to both protective/regenerative features and deleterious pathogenic processes. Therefore, defining the molecular and cellular processes that enhance, limit or resolve inflammation may lead to useful therapy. Muscle damage induces massive macrophage infiltration at the injury site, and macrophages are essential for muscle regeneration^1^,^2^,^3^,^4^. Indeed, partial depletion of mononuclear phagocytes impairs muscle regeneration, whereas reconstitution of myeloid lineage restores regeneration^2^,^5^,^6^. We previously showed that injured skeletal muscle recruits monocytes exhibiting inflammatory profiles that operate phagocytosis and rapidly convert to anti-inflammatory mononuclear phagocytes^3^. Aside from removing necrotic muscle debris, macrophages promote myogenic cell growth and differentiation by releasing growth factors and cytokines^7^,^8^, protect myogenic cells from apoptosis^9^ and participate in restoring perfusion by promoting collateral artery formation and angiogenesis^10^. In mice, infiltrating macrophages differentiate from circulating inflammatory Ly6C^high^ monocytes that co-express CCR2 and CX3CR1, two important chemokine receptors involved in monocyte motility^2^,^3^,^11^. In mice lacking CCR2 (*Ccr2*^−/−^) or its cognate ligand (*Ccl2*^−/−^), muscle regeneration and macrophage infiltration are impaired, and macrophage activation is modified^5^,^6^,^12^,^13^,^14^,^15^,^16^. By contrast, the role of CX3CR1 in muscle healing has not been defined yet, despite its importance in monocyte recruitment^17^,^18^, macrophage polarization^19^,^20^,^21^,^22^, cell survival^20^,^23^ and angiogenesis^24^,^25^.

In the skeletal muscle, recent works indicated that CX3CR1-negative resident macrophages localized in the normal epimysium/perimysium behave as sensors of pathological events, whereas exudate CX3CR1-expressing mononuclear phagocytes may provide immediate inflammatory response in the injured muscle and longstanding immune surveillance until full muscle restoration^26^. CX3CR1 is not recognized as a key molecule in resident macrophage ontogeny, although it plays a key role in kidney dendritic cells, brain microglial cells and intestinal macrophages colonization^27^,^28^,^29^. Few studies indicate that the survival of monocytes and tissue macrophages may rely on CX3CR1 as shown within the atherosclerotic plaques^30^ and in the liver^20^. More recently, CX3CR1 was identified as a regulator of renal macrophage proliferation^31^.

Chemokine (CX3C motif) ligand 1 (CX3CL1) also known as fractalkine or neurotactin is the only known member of the CX3C class of chemokines. It is unique because (i) it is expressed both as a membrane-bound and a soluble form and (ii) it binds specifically to its receptor CX3CR1. It is expressed by human and murine primary myoblasts in culture^32^,^33^. Whether the CX3CR1/CX3CL1 axis is involved in muscle healing through the recruitment of circulating monocytes or the activation of macrophages, and whether its relation to the CCL2/CCR2 axis is unequivocal, as in atherosclerosis^18^,^34^, or ambiguous, as in age-related macular degeneration (AMD)^35^, remains to be understood.

In this study, we investigate muscle regeneration and mononuclear phagocyte recruitment in a notexin-induced muscle injury model by comparing C57Bl/6 (wild type, WT), *Cx3cr1*^−/−^, *Ccl2*^−/−^ and *Cx3cr1*^−/−^ *Ccl2*^−/−^ mice. We show that CX3CR1 deficiency promotes muscle repair and rescues *Ccl2*^−/−^ mice from impaired muscle regeneration. These phenomena result not from altered mononuclear phagocyte infiltration in *Cx3cr1*^−/−^ mice but from functional modifications. Transcriptomic analysis were performed to compare muscle mononuclear phagocyte subsets between mice with efficient regeneration (WT, *Cx3cr1*^−/−^ and *Cx3cr1*^−/−^ *Ccl2*^−/−^ mice) and those with poor recovery (*Ccl2*^−/−^ mice). Among genes differentially expressed, Apolipoprotein E (*ApoE*) transcripts are downregulated in *Ccl2*^−/−^ mononuclear phagocyte subsets. Both ApoE administration and ApoE-proficient bone marrow (BM) transfer restore muscle regeneration in *Ccl2*^−/−^ mice. CX3CR1-ApoE-deficient BM transfer did not rescue CCL2-delayed muscle regeneration as effectively as CX3CR1-deficient ApoE-proficient transfer. Finally, ApoE enhances *in vitro* phagocytosis by macrophages, restores phagocytic activity in *Ccl2*^−/−^ mice, and its deficiency leads to incomplete necrotic myofiber removal. Our results suggest that targeting CX3CR1 expressed by mononuclear phagocytes might be useful in alleviating inflammation and promoting muscle regeneration after trauma.

## Results

### CX3CR1 deficiency promotes muscle healing after injury

**Figure 1:**
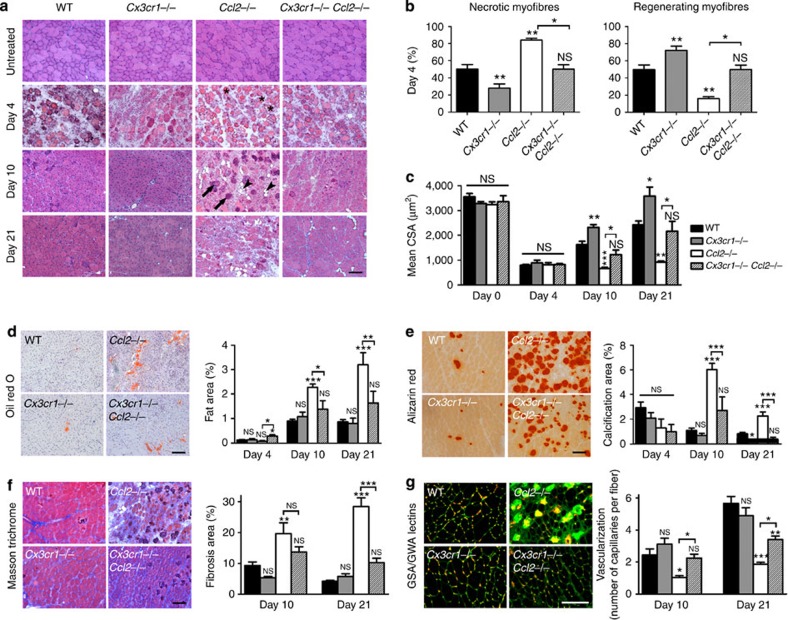
CX3CR1 deficiency promotes and rescues *Ccl2*^−/−^ muscle healing after injury. Muscle regeneration was assessed after notexin injury in WT, *Cx3cr1*^−/−^, *Ccl2*^−/−^ and *Cx3cr1*^−/−^ *Ccl2*^−/−^ mice on days 4, 10 and 21 post injury. (**a**) Haematoxylin/eosin staining of uninjured and regenerating muscle ( × 100 magnification, scale bar, 100 μm). Arrows indicate dense violet structures corresponding to calcified myofibers, and arrowheads mark intermuscular adipocytes (transparent monovacuolar cells). (**b**) Quantification of regeneration on day 4 post injury assessed by the percentage of necrotic (infiltrated or not) and regenerating (basophilic+centrally nucleated) myofibers, expressed as a percentage of the total number of myofibers. (**c**) Quantification of the mean cross-sectional area (CSA) of regenerating myofibers, by image J software. (**d**) Oil Red O staining of lipids within intermuscular adipocytes, (**e**) Alizarin Red staining of calcified myofibers, (**f**) Masson Trichrome staining of connective tissue and quantification of the different areas, by setting appropriate thresholds on image J software. Fat, calcification and fibrosis are expressed as percentages of the total injured area (between 60 and 90% of total muscle surface) and results are a mean of three different muscle cross-sections taken at each end and the middle of the muscle. (**d**, **e** and **f**: scale bar, 100 μm). (**g**) Vascularization was quantified on muscle cross-sections stained with wheat germ/*Griffonia simplicifolia* agglutinin (scale bar, 50 μm). Data are mean+s.e.m. of six mice per strain, which represent two distinct experiments. NS, not significant, *P<0.05, **P<0.01 and ***P<0.001 for each knock-out strain versus WT and for *Ccl2*^−/−^ versus *Cx3cr1*^−/−^ *Ccl2*^−/−^ mice.

We used a mouse model of notexin-induced muscle injury to analyse the muscle reparative process and mononuclear phagocyte recruitment. We show that CX3CR1 (Supplementary Fig. 1a,b) and its ligand CX3CL1 (Supplementary Fig. 1c) are both expressed during muscle regeneration with a maximum reached at day 4. Concomitantly, CCL2 was secreted at days 1 and 4 post injury (Supplementary Fig. 1d). To understand the roles of these chemokines and their functional relationship during muscle repair, we compared muscle regeneration events and mononuclear phagocyte recruitment between C57Bl/6 (WT), *Cx3cr1*^−/−^, *Ccl2*^−/−^ and *Cx3cr1*^−/−^ *Ccl2*^−/−^ mice. Notexin induced muscle necrosis (day 1, data not shown), which was followed by leukocyte infiltration (day 4), regenerating myofiber formation (day 10) and myofiber growth (day 21; Fig. 1a). Consistent with published data^16^, muscle regeneration was impaired in *Ccl2*^−/−^ mice compared with WT mice and was characterized by delays in necrotic fibre removal and myofiber regeneration (Fig. 1a,b and Supplementary Fig. 2), a smaller cross-sectional area (CSA) of regenerating myofibers (Fig. 1c), more intermuscular adipocytes (Fig. 1d), calcification (Fig. 1e) and fibrosis (Fig. 1f), as well as decreased neovascularization (Fig. 1g). CX3CR1 deficiency improved muscle regeneration: *Cx3cr1*^−/−^ mice had fewer necrotic and more regenerating myofibers on day 4 (Fig. 1a,b and Supplementary Fig. 2), higher myofiber CSA on days 10 and 21 post injury (Fig. 1c) and reduced intermuscular calcification (Fig. 1e). Strikingly, muscle regeneration in *Cx3cr1*^−/−^ *Ccl2*^−/−^ mice was better than in *Ccl2*^−/−^ mice and similar to that of WT mice for all the indicators studied; it thus enhanced the muscle regeneration improvement driven by the CX3CR1 deficiency (Fig. 1 and Supplementary Fig. 2). These results were unexpected, as CX3CR1 deficiency usually causes severe disorders in various inflammatory models, associated with cell death^36^, fibrosis development^20^, delayed wound healing and neovascularization^24^,^25^. The observation of the rescued phenotype in both male and female double-deficient mice (Supplementary Fig. 3) but not in *Cx3cr1*^−/−^ *Ccr2*^−/−^ compared with *Ccr2*^−/−^ mice (Supplementary Fig. 4) suggests that a minimum level of monocyte infiltration is required to compensate for the muscle regeneration impairment (monocyte recruitment is more severely impaired in *Ccr2*^−/−^ than *Ccl2*^−/−^ mice^12^). CX3CR1 deficiency thus appears to promote muscle repair and rescue *Ccl2*^−/−^ mice from impaired muscle regeneration.

### CCL2 but not CX3CR1 deficiency alters macrophage infiltration

**Figure 2:**
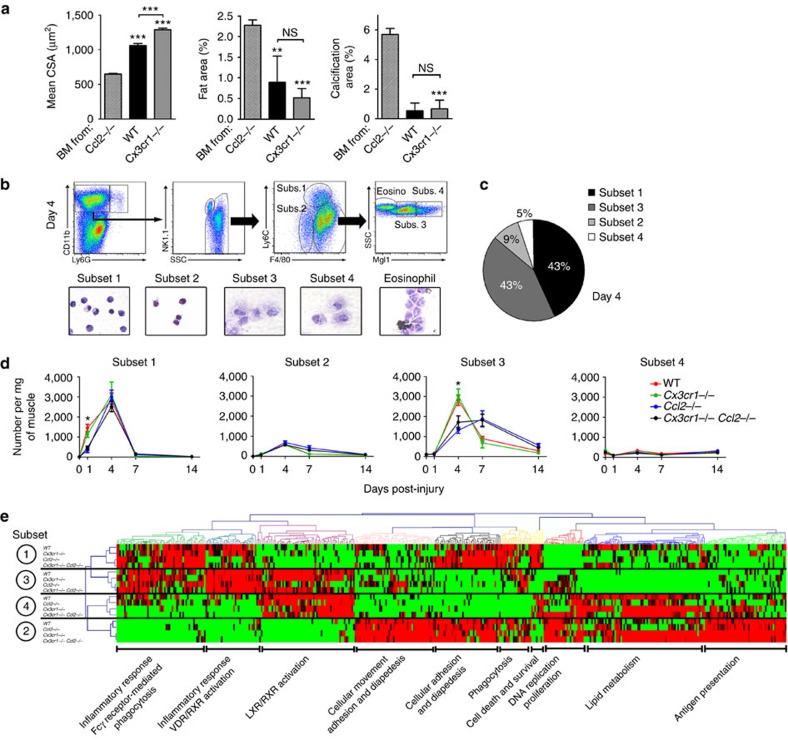
CCL2 but not CX3CR1 deficiency affects macrophage recruitment in muscle. (**a**) Analysis of muscle regeneration in chimeric *Ccl2*^−/−^ mice engrafted with bone marrow from *Ccl2*^−/−^, WT and *Cx3cr1*^−/−^ mice, by measuring the mean regenerating myofiber CSA and the areas of intermuscular adipocyte and of calcified myofibers on day 10 post injury. ***P<0.001 for *Ccl2*^−/−^ mice engrafted with bone marrow from WT or *Cx3cr1*^−/−^ mice versus *Ccl2*^−/−^ mice engrafted with *Ccl2*^−/−^ bone marrow. Data are mean of a single experiment+s.e.m. of three mice per strain. (**b**) Gating strategy for macrophages on day 4 post injury. Macrophages expressed CD11b, F4/80 and Mgl1 but not Ly6G or NK1.1. Four subsets of cells corresponding to this phenotype were identified; a subset of CD11b^+^ F4/80^+^ Ly6C^low^ Mgl1^−^ cells, with morphology typical of eosinophils, was excluded from the analysis. (**c**) Proportion of each macrophage subset on day 4 post injury. (**d**) Kinetics of the four macrophage subsets during muscle regeneration in WT, *Cx3cr1*^−/−^, *Ccl2*^−/−^ and *Cx3cr1*^−/−^ *Ccl2*^−/−^ mice. *P<0.05 for *Ccl2*^−/−^ and *Cx3cr1*^−/−^ *Ccl2*^−/−^ strains versus WT. Data are mean of two to four distinct experiments±s.e.m. of at least six mice per strain per time point. (**e**) The four macrophage subsets from injured muscle were sorted by flow cytometry on day 4 post injury according to the gating strategy described in (**b**). RNA from one cell-sorting experiment, representing a pool of three mice per strain, was amplified and hybridized on the Illumina whole mouse genome oligo microarrays (WG6). Hierarchical clustered heat map of macrophage subsets 1, 2, 3 and 4 based on the gene expression of ten different clusters. Red: increased expression, green: decreased expression.

To identify the cellular origin of the muscle regeneration rescue observed in *Cx3cr1*^−/−^ *Ccl2*^−/−^ mice, we performed BM transplantation experiments in sub-lethally irradiated *Ccl2*^−/−^ mice. The control graft produced strongly improved muscle regeneration on day 10 post injury, characterized by increased regenerating myofiber CSA and decreased intermuscular fat accumulation and calcification (Fig. 2a). BM transfer from *Cx3cr1*^−/−^ mice amplified muscle regeneration rescue and increased the regenerating myofiber CSA. This indicates that the muscle regeneration improvement observed in *Cx3cr1*^−/−^ mice relied on BM-derived cells.

As both CX3CR1 and CCL2 are involved in mononuclear phagocyte trafficking, we hypothesized that modulation of mononuclear phagocyte recruitment in the injured muscle might account for the improved regeneration in *Cx3cr1*^−/−^ mice and rescue of *Cx3cr1*^−/−^ *Ccl2*^−/−^ mice. We assessed mononuclear phagocyte infiltration throughout the injured muscle by flow cytometry (Fig. 2b) and identified four distinct populations (subsets 1–4) expressing typical mononuclear phagocyte phenotypic markers (CD11b^+^ Ly6G^−^ NK1.1^−^ F4/80^+^ Mgl1^+^; Fig. 2b and Supplementary Fig. 5). Subset 1 (F4/80^low^ Ly6C^high^) has bean-shaped nuclei and may correspond to infiltrating Ly6C^high^ blood monocytes. Subset 2 (F4/80^low^ Ly6C^low^) expressed dendritic cell markers, such as CD11c and IAb (major histocompatibility complex molecules-class II), and CD64 (FcγRI) at lower levels than the other three subsets. Cells from subsets 3 and 4 were larger and had typical macrophage morphology; subset 3 (F4/80^high^ Ly6C^low^ Mgl1^low^) expressed lower levels of M2-type macrophage markers (such as Mgl1 and CD206) than subset 4 (F4/80^high^ Ly6C^low^ Mgl1^high^; Fig. 2b and Supplementary Fig. 5). Cells from subsets 1 and 3 were the main mononuclear phagocyte subsets (about 86%) in muscle at the peak of inflammation, on day 4 post injury (Fig. 2c). In a previous study^2^, we showed that Ly6C ^high^ monocytes in the muscle are recruited from the blood and are non-dividing cells. They switched their phenotype to Ly6C^low^ mononuclear phagocytes and actively proliferated *in situ*, contributing to the large amount of mononuclear phagocytes observed in the muscle at day 4 post injury.

Subset 1 was found in significantly lower numbers on day 1 (70% reduction, *P*<0.0006) as was subset 3 on day 4 (48% reduction, *P*<0.0001) in injured muscles from *Ccl2*^−/−^ and *Cx3cr1*^−/−^ *Ccl2*^−/−^ compared with WT and *Cx3cr1*^−/−^ mice (Fig. 2d). This finding confirms that monocyte/macrophage recruitment requires CCL2 (ref. 12). Conversely, CCL2 and CX3CR1 deficiency did not or only slightly affected the proportions of subsets 2 and 4. The greater impairment of the recruitment of subsets 1 and 3 in the injured muscle of *Ccr2*^−/−^ mice (Supplementary Fig. 6) suggests that their presence in the lesion is necessary for the CX3CR1-dependent compensatory effect. Strikingly, the infiltration kinetics of all mononuclear phagocyte subsets of *Ccl2*^−/−^ and *Cx3cr1*^−/−^ *Ccl2*^−/−^ mice, as for WT and *Cx3cr1*^−/−^ mice, were superimposable (Fig. 2d) despite strongly different pathophysiological conditions. The apparent lack of association between the muscle regeneration rescue observed in *Cx3cr1*^−/−^ *Ccl2*^−/−^ mice and any change in the number of monocytes infiltrating/macrophages proliferating in the injured muscle suggests that the process allowing muscle recovery in *Cx3cr1*^−/−^ mice may be related to mononuclear phagocyte functions. We next investigated the role of BM-derived macrophages from the various mice strains regarding myogenic precursor cells (MPCs) using *in vitro* assays. No differences were observed in MPC proliferation or fusion (Supplementary Fig. 7). Neither CX3CR1 nor CCR2 were detected on satellite cells^13^, on myoblasts or on regenerating myofibers (data not shown).

To address more globally the mononuclear phagocyte functions that may promote muscle regeneration, we performed a genome-wide microarray analysis of the four mononuclear phagocyte subsets from WT, *Cx3cr1*^−/−^*, Ccl2*^−/−^ and *Cx3cr1*^−/−^ *Ccl2*^−/−^ muscles on day 4 post injury (GSE73473). One-way analysis of variance analysis identified 434 genes (*P*<0.05) that distinguished the four subsets (Fig. 2e). Using hierarchical clustering and pathway analysis, we identified 10 clusters that confirmed the original identity and functions of each subset. Because muscle regeneration was not rescued in *Cx3cr1*^−/−^ *Ccr2*^−/−^ mice (Supplementary Fig. 4) and muscle infiltration of subsets 1 and 3 was fully abrogated only in *Ccr2*^−/−^ mice (Supplementary Fig. 5), we postulated that these subsets were mainly responsible for this rescued phenotype and sought to identify in them the genes that promote healing.

### ApoE production is adversely regulated by CCL2 and CX3CR1

**Figure 3:**
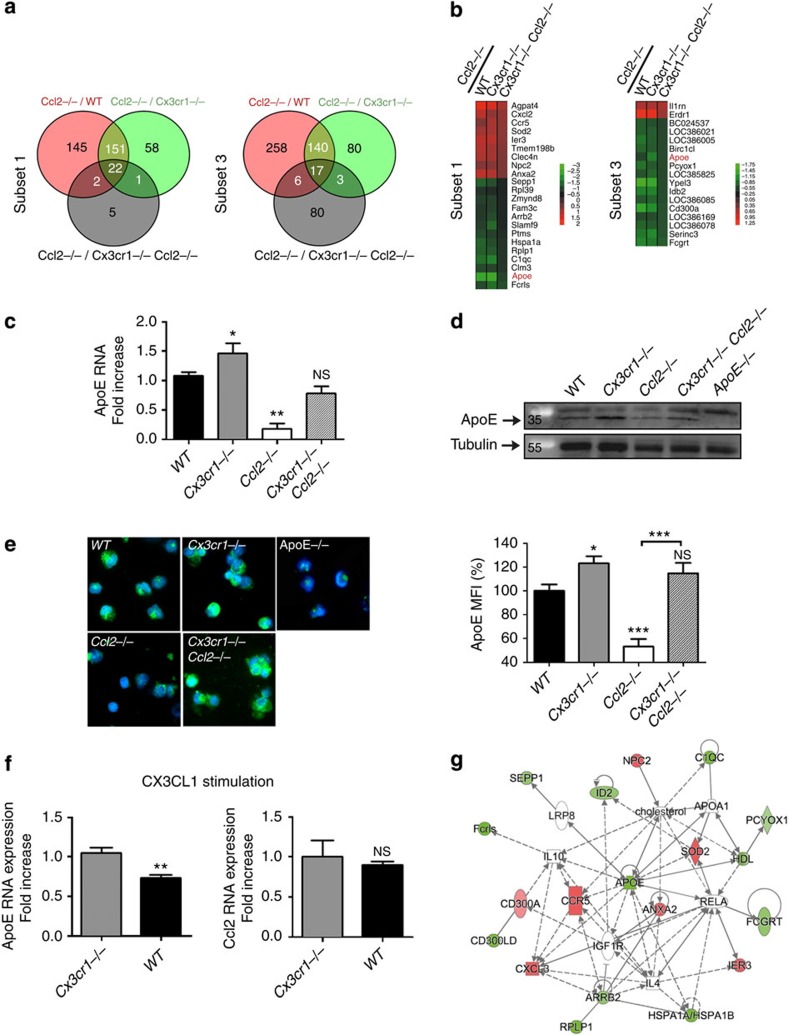
ApoE macrophage production is adversely regulated by CCL2 and CX3CR1. (**a**) Venn diagrams illustrating the comparisons between the set of genes significantly differentially regulated in cell subsets 1 and 3 from *Ccl2*^−/−^ versus WT (in red), *Cx3cr1*^−/−^ (in green) and *Cx3cr1*^−/−^ *Ccl2*^−/−^ (in grey), mice. (**b**) Heat maps representing the positive (red) and negative (green) log2 ratio of gene expression in subsets 1 (22 genes) and 3 (17 genes) from *Ccl2*^−/−^ mice compared with WT, *Cx3cr1*^−/−^ and *Cx3cr1*^−/−^ *Ccl2*^−/−^ mice. ApoE expression in the global pool of mononuclear phagocytes extracted from injured muscle though magnetic columns (CD11b^+^ Ly6G^−^ NK1.1^−^) was analysed for each mouse strain on day 4 post injury by: (**c**) Real time-PCR (data are a mean+s.e.m. of three experiments). (**d**) Western blotting (one experiment representative of two; first lane: size marker in kDa), (**e**) immunolabelling and quantification of mean fluorescent intensity (MFI), with the appropriate threshold using image J software. (Data are a mean+s.e.m. of at least five fields counted (magnification of × 40) from two cell sorting experiments.) NS, not significant, *P<0.05, **P<0.01 and ***P<0.001 for each knock-out strain versus wild type. (**f**) Modifications in ApoE and Ccl2 RNA expression induced after CX3CL1 (100 nM) *in vitro* stimulation during 16 h was analysed by real-time PCR in the global pool of mononuclear phagocytes extracted from WT mice, compared with *Cx3cr1*^−/−^. Data are a mean+s.e.m. for six mice per strain in three distinct experiments; NS, not significant, **P<0.01. (**g**) Network diagram showing the links between most of the genes differentially up- (red) or downregulated (green) in *Ccl2*^−/−^ mononuclear phagocytes compared with *Cx3cr1*^−/−^ *Ccl2*^−/−^ mononuclear phagocytes, inflammatory response, endocytosis signalling, LXR/RXR activation and lipid metabolism (Ingenuity Pathway analysis).

Comparison of the mononuclear phagocyte transcriptomic profiles from mice with poor regenerative abilities (*Ccl2*^−/−^) and from mice with effective muscle healing (WT, *Cx3cr1*^−/−^ and *Cx3cr1*^−/−^ *Ccl2*^−/−^) identified 22 and 17 genes significantly differentially expressed in mononuclear phagocyte subsets 1 and 3, respectively (Fig. 3a,b). Among them, only ApoE transcripts were downregulated in both *Ccl2*^−/−^ subsets 1 and 3. We confirmed severely lower ApoE expression in macrophages isolated from injured muscle of *Ccl2*^−/−^ mice at both RNA (Fig. 3c) and protein levels (Fig. 3d,e and Supplementary Fig. 8). In accordance with the microarray data, ApoE expression was restored in *Cx3cr1*^−/−^*Ccl2*^−/−^ mice. Moreover, we found a higher expression of ApoE in mononuclear phagocytes from CX3CR1-deficient compared with CX3CR1-proficient mice (Fig. 3c–e). We also showed that addition of CX3CL1 on WT macrophages *in vitro* suppressed ApoE expression by about 30%, without affecting CCL2 expression (Fig. 3f). These results reveal that CX3CL1/CX3CR1 axis is also directly involved in the regulation of ApoE expression.

Interestingly, most of the dysregulated genes in mononuclear phagocytes from *Ccl2*^−/−^ versus WT, *Cx3cr1*^−/−^ and *Cx3cr1*^−/−^ *Ccl2*^−/−^ mice can be linked in a network in which ApoE is central and related to inflammatory response, endocytosis signalling and the LXR/RXR activation pathway involved in the regulation of inflammation and lipid metabolism^37^ (Fig. 3g). We previously published a functional link between ApoE and CX3CR1 in AMD^38^. Sub-retinal macrophages in AMD patients as well as in sub-retinal inflammation observed in *Cx3cr1*^−/−^ mice express high levels of ApoE. Previous studies indicate that ApoE is an immunomodulatory agent that polarizes macrophages in a M2 anti-inflammatory phenotype^39^. We previously showed that M2 macrophages are associated with myofiber growth and myoblast differentiation and fusion^3^. In addition, ApoE can suppress the pro-inflammatory response mediated by microbial stimuli *in vivo*^40^,^41^. Furthermore, the delay in skeletal muscle healing in *ApoE*^−/−^ mice following hindlimb ischaemia-reperfusion^42^ suggests that this gene plays a major role in the muscle regeneration process.

### ApoE favours phagocytosis and muscle regeneration

**Figure 4:**
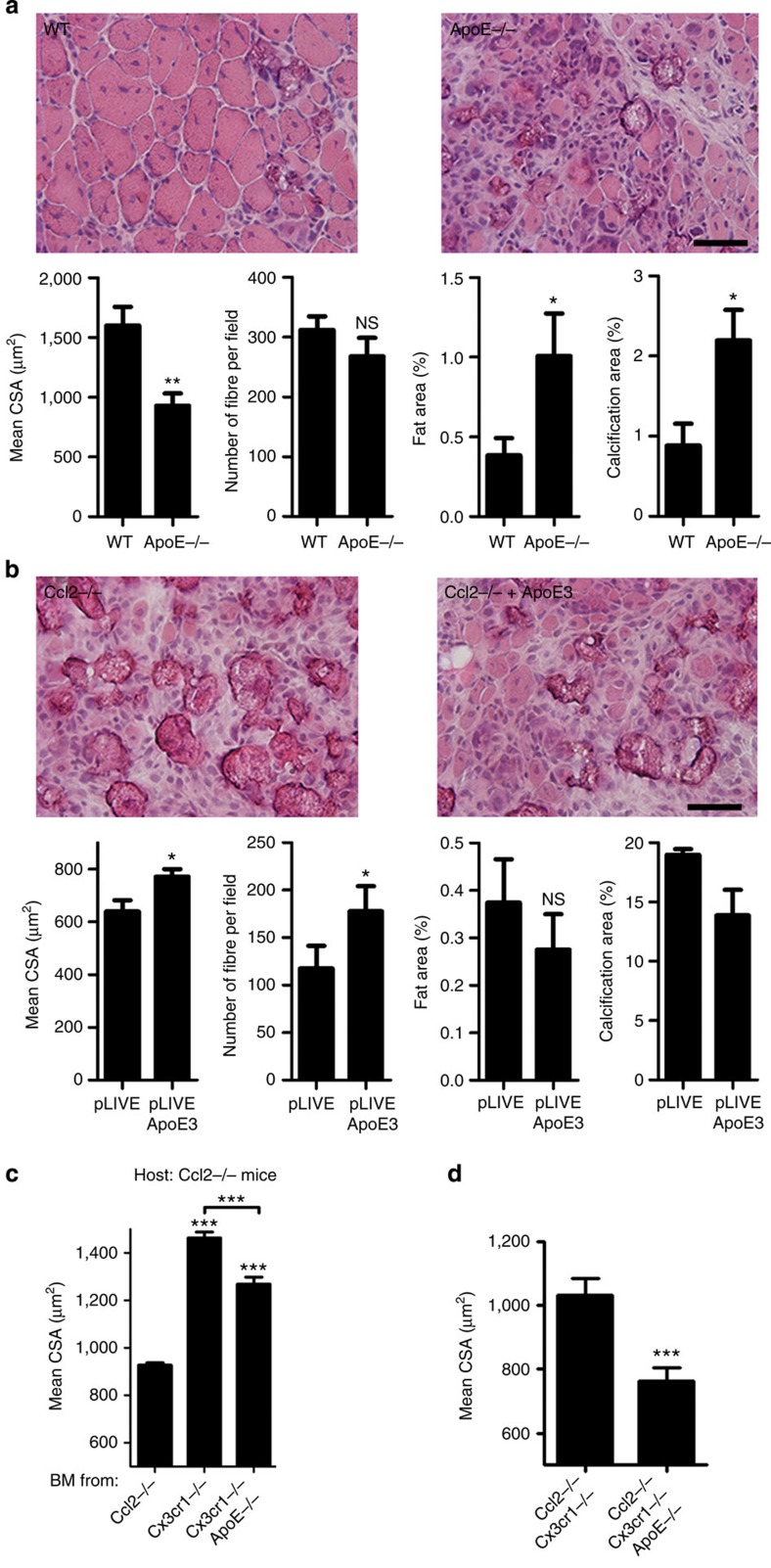
Macrophage ApoE production improves skeletal muscle regeneration. (**a**) Analysis of muscle regeneration of *ApoE*^−/−^ (*n*=11) versus WT mice (*n*=7) on day 10 post injury, by measurement of mean CSA of regenerating myofibers, myofiber density and areas of fat and calcification. Data are mean+s.e.m. of two distinct experiments. (**b**) Analysis on day 10 post injury of muscle regeneration in *Ccl2*^−/−^ mice, transfected (*n*=3) or not (*n*=3) with a pLIVE plasmid encoding for human ApoE3, by measurement of mean CSA of regenerating myofibers, myofiber density and areas of fat and calcification. In this experimental system, systemic expression of ApoE3 was detected for at least 8 days after transfection (data not shown). Data are mean+s.e.m. of a single experiment. Scale bar, 100 μm. (**c**) Analysis of muscle regeneration in chimeric *Ccl2*^−/−^ mice engrafted with *Ccl2*^−/−^ (*n*=5), *Cx3cr1*^−/−^ (*n*=5) or *Cx3cr1*^−/−^ *ApoE*^−/−^ (*n*=4) bone marrow, by measurement of the mean CSA of regenerating myofibers on day 21 post injury. (**d**) Analysis of muscle regeneration in *Cx3cr1*^−/−^ *Ccl2*^−/−^ mice (*n*=4) and *Cx3cr1*^−/−^ *Ccl2*^−/−^ *ApoE*^−/−^ mice (*n*=4). Data are mean+s.e.m.; NS, not significant, *P<0.05, **P<0.01 and ***P<0.001.

Histological analysis of injured muscle from *ApoE*^−/−^ mice 10 days after injury revealed strongly impaired muscle regeneration, with a markedly lower CSA of regenerating myofiber and increased intermuscular fat and calcification (Fig. 4a). These results are consistent with previous reports that *ApoE*^−/−^ mice develop calcification and accumulate lipid-filled foam cells within atheromatous plaque when fed a high-fat diet^43^,^44^. Using hydrodynamic-based *in vivo* transfection, we next showed that systemic expression of human ApoE3 improved muscle repair in *Ccl2*^−/−^ mice (Fig. 4b). In addition, *Cx3cr1*^−/−^ *ApoE*^−/−^ bone-marrow transfer in irradiated *Ccl2*^−/−^ mice did not promote myofiber growth as effectively as *Cx3cr1*^−/−^ bone-marrow transfer (+33% myofiber CSA versus +58%; Fig. 4c). We also observed high reduction of myofiber growth in *Cx3cr1*^−/−^ *Ccl2*^−/−^ *ApoE*^−/−^ mice compared with *Cx3cr1*^−/−^ *Ccl2*^−/−^ mice (Fig. 4d). These results demonstrate that the impairment of muscle regeneration in *Ccl2*^−/−^ mice depends at least partially on the inability of muscle mononuclear phagocytes to produce enough ApoE and that CX3CR1 deficiency promotes and rescues *Ccl2*^−/−^ muscle healing by regulating ApoE production by macrophages.

**Figure 5:**
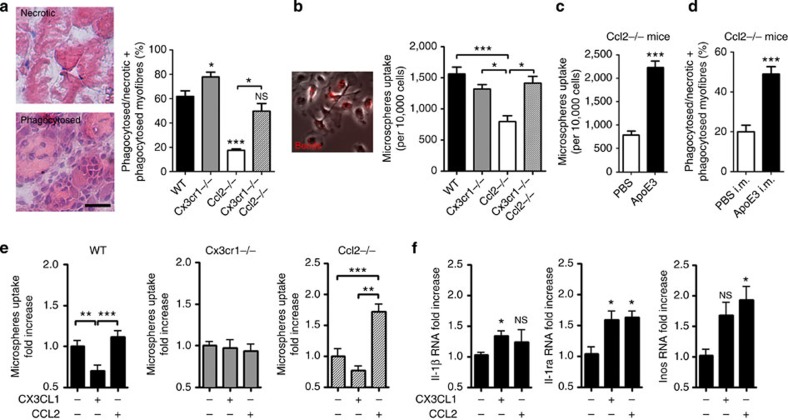
Macrophage ApoE production enhances muscle debris phagocytosis. (**a**) Quantification of *in vivo* phagocytosis, by assessment of the numbers of infiltrated necrotic myofibers versus total necrotic myofibers infiltrated or not on day 4 post injury. Scale bar, 100 μm. Data are means+s.e.m. of six mice per strain in two independent experiments. (**b**) Phagocytosis activity of mononuclear phagocytes extracted from injured-muscle of WT, *Cx3cr1*^−/−^, *Ccl2*^−/−^ and *Cx3cr1*^−/−^ *Ccl2*^−/−^ mice on day 4 post injury was assessed by incubating muscle cells for 4 h with red fluorescent microspheres. This parameter is expressed as the number of cells that engulfed at least one bead per 10,000 cells. Results are means+s.e.m. of six mice in three independent experiments. (**c**) Phagocytosis activity of *Ccl2*^−/−^ muscle mononuclear phagocytes in the absence or presence of exogenous human APOE3 protein. Results are mean±s.e.m. of five mice in two independent experiments. (**d**) Quantification on day 4 post injury of *Ccl2*^−/−^ myofiber removal in the absence (*n*=2) or presence (*n*=4) of exogenous human APOE3 protein. (**e**) Phagocytosis activity of mononuclear phagocytes from injured-muscle of WT, *Cx3cr1*^−/−^ and *Ccl2*^−/−^ mice on day 4 post injury was assessed 16 h after incubation of muscle cells with CX3CL1 or CCL2 chemokines. The numbers of muscle cells that engulfed at least one fluorescent microsphere were compared between treated and untreated cells for all mice strains. (**f**) Effect of CX3CL1 and CCL2 on IL-1, IL-1 receptor antagonist (Ra) and iNOS (nitric oxide synthase) expression was measured by real-time PCR in mononuclear phagocytes extracted from WT mice incubated during 16 h with CX3CL1 (100 nM) or CCL2 (100 nM), and compared with untreated cells. Data are mean+s.e.m. of six mice per strain in three independent experiments. NS, not significant, *P<0.05, **P<0.01 and ***P<0.001.

Because macrophage phagocytic activity is impaired in *ApoE*^−/−^ (Supplementary Fig. 9) and improved in *Cx3cr1*^−/−^ mice^21^,^45^, we hypothesized that improved muscle regeneration in *Cx3cr1*^−/−^ mice and rescue in *Cx3cr1*^−/−^ *Ccl2*^−/−^ mice might be linked to increased phagocytosis, and indeed we measured more phagocytic activity in muscles from *Cx3cr1*^−/−^ compared with WT mice (Fig. 5a). The better necrotic myofiber removal in *Cx3cr1*^−/−^ *Ccl2*^−/−^ compared with *Ccl2*^−/−^ mice (for an equivalent number of infiltrated mononuclear phagocytes) suggests that CX3CR1-deficient mononuclear phagocytes have higher phagocytic activity. *Ex vivo* experiments confirmed that muscle macrophages from *Cx3cr1*^−/−^ and *Cx3cr1*^−/−^ *Ccl2*^−/−^ mice engulfed fluorescent microspheres more effectively than those from *Ccl2*^−/−^ animals (Fig. 5b). Moreover, addition of exogenous APOE3 improved both phagocytic activity of *Ccl2*^−/−^ muscle macrophages *in vitro* (Fig. 5c) and necrotic myofibre removal in *Ccl2*^−/−^ mice (Fig. 5d). Addition of CCL2 restored phagocytic activity of *Ccl2*^−/−^ macrophages (Fig. 5e, right panel) but not that of WT and *Cx3cr1*^−/−^ macrophages. A reduction in WT macrophage phagocytic capacity was observed after incubation with CX3CL1 (Fig. 5e, left panel). Finally, the addition of CX3CL1 and CCL2 on WT macrophage-enriched muscular cell suspensions triggered similar expression of molecules involved in inflammatory response such as IL-1β, IL-1RA (IL-1 receptor antagonist) and iNOS (inducible nitric oxide synthase; Fig. 5f). Altogether, our results suggest that impaired muscle healing in *Ccl2*^−/−^ mice is linked to decreases in monocyte/macrophage recruitment and function, notably phagocytosis.

## Discussion

Skeletal muscle regeneration is a complex series of events, in which monocytes/macrophages play a major part in the healing process. Chemokines and their receptors orchestrate their distribution and their functions to ensure muscle regeneration. CX3CR1 and CCL2 deficiencies affect post-lesion blood monocytosis differently: CX3CR1 deficiency is associated with a reduction in Ly6C^low^ monocytes, whereas CCL2 deficiency affects the number of Ly6C^low^ and Ly6C^high^ monocytes (Supplementary Fig. 10). Because Ly6C^low^ blood monocytes have no role in the muscle regeneration process^3^,^46^, their lack may not impair this process. Few groups including Saederup *et al*.^47^ and our group^18^ have shown additive or synergistic effects of CCR2 and CX3CR1 axis on monocyte recruitment or macrophage accumulation. CCR2 and CX3CR1 axis may also lead to the opposite effect, and more specifically CX3CR1 deficiency may promote CCR2-dependent microglial cell accumulation in the retina^35^. Here we could not detect any changes in monocyte recruitment or in macrophage accumulation consistently with our previous work showing that monocyte recruitment in the injured muscle was exclusively dependent on CCR2 axis^3^. In these conditions, CX3CR1 may only affect macrophage functions, not monocyte recruitment or macrophage proliferation.

CX3CR1 is an essential pleiotropic chemokine receptor involved in various biological functions, including cell chemotaxis, apoptosis, cell activation/polarization, organ development and neovascularization. Its deficiency is considered deleterious in a large variety of acute inflammatory responses where it exacerbates inflammation^20^,^24^,^36^. In chronic diseases, such as atherosclerosis, CX3CR1 deficiency also limits monocyte recruitment and macrophage accumulation at inflammatory sites leading to reduced atherosclerosis^18^,^48^,^49^. In contrast, CX3CR1 presence is somewhat protective in AMD^50^. The role of CX3CR1 appears to be different depending on the inflammatory model. Our results show for the first time that CX3CR1 deficiency can improve wound healing in an acute injury model without affecting monocyte recruitment or macrophage accumulation. In the mdx mice, a chronic model of muscle injury (a murine model for Duchenne muscular dystrophy), previous studies using gene expression microarrays have demonstrated that dystrophic muscles are characterized by an inflammatory ‘molecular signature'^51^,^52^, in which CC chemokines are prominent and among which CCL2 and its receptor CCR2 are highly expressed^53^,^54^. There is no evidence of CX3CR1/CX3CL1 expression in this context and the role of CX3CR1 has not yet been investigated. In a previous work^9^, we showed that four molecular systems including CX3CR1/CX3CL1 are involved in MPC apoptosis inhibition driven by macrophages *in vivo* and *in vitro*. Although apoptosis induction is fairly easy to achieve *in vitro* (through the incubation with various pro-apoptotic drugs), the MPC apoptosis events *in vivo* are rather rare, with apoptotic MPC representing only a very small number of cells in the myoblast population. In contrast, phagocytosis events during muscle repair are ubiquitous, and it is more likely that macrophages will engulf apoptotic MPC *in vivo* than protect them through the CX3CR1/CX3CL1 molecular system.

**Figure 6:**
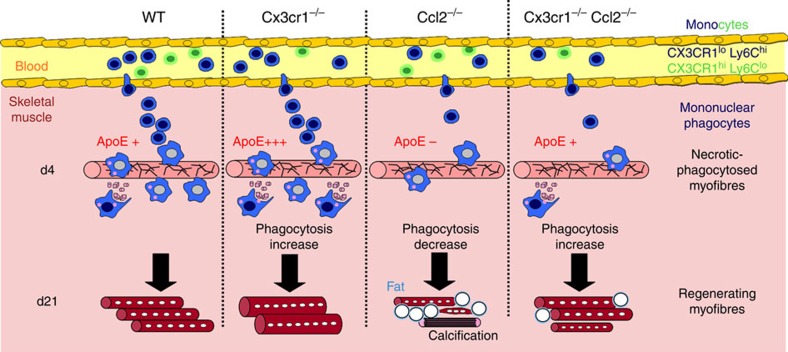
Macrophage infiltration and function during muscle regeneration. Diagram depicting macrophage infiltration and functions during muscle regeneration in WT, *Cx3cr1*^−/−^, *Ccl2*^−/−^ and *Cx3cr1*^−/−^ *Ccl2*^−/−^ mice.

Figure 6 summarizes the main findings of the work and proposes a model of action of macrophages in muscle regeneration via ApoE production. We demonstrated that CX3CR1 deficiency is associated with increased removal of necrotic myofibers, as it is with the phagocytosis of amyloid-beta deposits in the brain of a murine model of Alzheimer's disease^21^. Similarly, CX3CR1-deficient mononuclear phagocytes overexpressed ApoE protein, which is known to stimulate macrophage phagocytosis and dampen inflammatory response^39^,^45^. Moreover, a recent study^38^ demonstrated a link between ApoE and CX3CR1 in AMD. Sub-retinal macrophages in AMD patients as well as in sub-retinal inflammation observed in *Cx3cr1*^−/−^ mice express high levels of ApoE. In addition, ApoE deletion in *Cx3cr1*^−/−^ mice prevents pathogenic- and stress-induced subretinal macrophage accumulation. Here we showed that the exogenous administration of ApoE enhanced CCL2-deficient mononuclear phagocyte phagocytic activity. These results suggest that defective CCL2-dependent monocyte recruitment may be compensated by CX3CR1 deficiency through the modulation of ApoE-dependent macrophage phagocytic activity. Therefore, therapeutic intervention to improve mononuclear phagocyte functions by targeting the CX3CR1 chemokine axis might control inflammation and enhance muscle regeneration in response to injury.

## Methods

### Mice

Experiments were conducted on adult female and male mice (8–16 weeks old) of the following strains (all on a C57Bl/6J genetic background): C57Bl/6J (WT; purchased from Janvier), *Cx3cr1*^−/−^ (ref. 55), *Ccl2*^−/−^ (#004434, JAX), *Ccr2*^−/−^ (#004999, JAX), *ApoE*^−/−^ (#002052, JAX), *Cx3cr1*^*gfp/+*^ (#008451, JAX) were intercrossed to obtain *Cx3cr1*^−/−^ *Ccl2*^−/−^, *Cx3cr1*^−/−^ *ApoE*^−/−^, *Cx3cr1*^−/−^ *Ccl2*^−/−^ *ApoE*^−/−^ *and CX3CR1*^*gfp/gfp*^ *ApoE*^−/−^ as described in ref. 18. Mice were bred and maintained in our specific pathogen-free animal facility according to institutional guidelines and used according to the French legislation (no. A-75-1315). All experiment protocols were approved by the local ethics committee and validated by the Service Protection et Santé Animales, Environnement (no. A-75-2065).

### Muscle injury

Muscle injury was caused by intramuscular injection of 10 μl of notexin (25 μg ml^−1^ in PBS; Latoxan) into the tibialis anterior (TA) muscle. Muscle and fascia were harvested together for analysis at different time points post injury (1, 4, 7, 10, 14, 21 days). In some experiments, a pLIVE plasmid encoding for human ApoE3 was injected into mouse tail veins 6 h before muscle injury.

### Histological analysis

TA muscles were removed, snap frozen in nitrogen-chilled isopentane and kept at −80 °C until use for histological analysis. Cryosections of 7 μm thick were prepared for various types of histological staining. Muscle regeneration was analysed quantitatively for the entire injured area (between 60 and 90% of the entire muscle). The percentages of necrotic and regenerating myofibers as well as the CSA of the regenerating myofibers, an established indicator, were quantified on haematoxylin/eosin-stained muscle sections recorded with an Olympus BX51 at × 10 magnification, connected to an evolution VF camera (medicaCybernetics). We used image J software to count three fields always chosen in the same overall injured area (representing 660±150 myofibers) for at least six different mice analysed per time point. Regenerating myofiber density was assessed in the same fields and is expressed as the number of regenerating myofibers per field. Phagocytosis was assessed on day 4 post injury on muscle cross-sections stained with haematoxylin/eosin by counting the ratio of infiltrated to non-infiltrated necrotic myofibers. Vascularization was quantified on muscle cross-sections stained with wheat germ/*Griffonia simplicifolia* agglutinin. We counted 8±1 fields (at × 20 magnification) chosen randomly over the entire injured area and representing 485±160 myofibers, in three different mice. Areas of fat, calcification and fibrosis were evaluated over the entire injured area of three muscle cross-sections at levels spaced 900 μm apart, stained, respectively, with Oil Red O, Alizarin Red and Masson Trichrome and recorded with a Nikon AZ100 macroscope connected to a DS-Ri1 camera (Nikon). Appropriate thresholds were set with image J software to evaluate these areas. Each parameter was studied on days 4 (except fibrosis), 10 and 21 post injury in at least six different mice.

### Isolation of leukocytes and macrophages from muscle

Mice were flushed with 10 ml of PBS to remove blood. Muscles and fascia were weighed together, minced and digested twice for 45 min each time in DMEM containing collagenase B 0.2% (Roche Diagnostics) and trypsin-EDTA 0.2% at 37 °C. The resulting homogenate was filtered through a 70-μm cell strainer. In some experiments, macrophages were isolated from muscle cell suspension using MACS technology (Miltenyi Biotec). A negative selection using PE-Ly6G and PE-NK1.1 antibodies and anti-PE magnetic beads (to remove neutrophils and NK cells) was followed by a positive selection of macrophages with magnetic beads coupled with CD11b antibodies.

### Flow cytometry analysis and cell sorting

Cell suspensions were incubated with anti-mouse FcγII/III receptor (clone 2.4G2, BD Biosciences, 1:100) for 10 min at 4 °C in FACS buffer (Ca^2+/^Mg^2+^-free PBS with 0.5% BSA) and then stained with the following fluorescent-conjugated anti-mouse antibodies: V450-Ly6G (clone 1A8, BD Biosciences, 1:100), FITC-CD11c (clone HL3, BD Biosciences, 1:100), PE-Cy7-NK1.1 (clone PK136, BD Biosciences, 1:100), APC-Cy7-Ly6C (clone AL-21, BD Biosciences, 1:100), PE-CD64 (clone X54-5/7.1, BD Biosciences, 1:100), APC-IAb (major histocompatibility complex molecules-class II, clone AF6-120-1, BD Biosciences, 1:200), PE-SiglecF (clone E50-2440, BD Biosciences, 1:100), PE-CD206 (clone MR5D3, BD Biosciences, 1:100), biotinylated anti-Mgl1 (MCA2392B, Serotec, 1:100), PE-CCR2 (clone 475301, R&D Systems, 1:100), PerCP-Cy5.5-CD11b (clone M1/70, ebiosciences, 1:500) and APC-F4/80 (clone BM8, ebiosciences, 1:150). A PE-streptavidin (1:1,500, BD Biosciences) was used to reveal Mgl1 expression. Cells were analysed on a FACS Canto II (BD Biosciences) with Diva and FlowJo softwares. Sorting of macrophage cell subsets was performed on a FACSAria II cell sorter (BD Biosciences).

### Microarray analysis, normalization, data set analysis

The four subsets of mononuclear phagocytes were sorted from the injured muscle by flow cytometry on day 4 post injury with the gating strategy showed in Fig. 2. RNA was amplified with the Ovation Pico WTA system V2 (NuGEN) and the resulting complementary DNA (cDNA) was hybridized on Illumina whole mouse genome oligo microarrays (WG6 V2.0). Array data were analysed with Genome studio (Illumina) and MeV software. Filter on background signals and quantile normalization were applied, and only probe sets showing significance (*P*-value<0.05) in 8 of the 16 samples were selected. One-way analysis of variance (*P*<0.01) with subsequent hierarchical clustering was performed on median centred log2 intensity of the resulting 2,472 probes and identified 434 genes that discriminated between the four cell subsets and spread into 10 clusters. Genes showing differential expression in *Ccl2*^−/−^ mice compared with WT, *Cx3cr1*^−/−^ and *Cx3cr1*^−/−^ *Cc2*^−/−^ were selected according to Eisen *et al*.^56^ and comparisons are presented with Venn diagrams (http://bioinfogp.cnb.csic.es/tools/venny/index.html). Ingenuity Pathway Analysis (IPA; Ingenuity Systems Inc.) software was used to identify metabolic and signalling pathways.

### Quantitative RT–PCR

Total RNA from cell lysates of injured muscles was extracted using the RNeasy Mini Kit (Qiagen), according to the manufacturer's instructions. Reverse transcription was performed on 10 ng of RNA using SuperScript VILO cDNA Synthesis Kit (Invitrogen). cDNA were quantified by qPCR using Power SYBR Green PCR Master Mix (Life Technologies) and ApoE, Cx3cr1, Ccl2, Il-1β, Il-1Ra (receptor antagonist), iNOS primers were added at 250 nM each in a final volume of 20 μl (in duplicate for each sample). The sequences of primers used are as follows: *ApoE*-F: 5′- CCTGAACCGCTTCTGGGATT -3′, *ApoE*-R:5′- CGTAGATCCTCCATGTCGGC -3′, *Cx3cr1*-F: 5′- TTCAATCCCAAGGCCCTGTC -3′, *Cx3cr1*-R: 5′- GTGCAAGCAACAGAGTTGGG -3′, *Il1β*-F: 5′- CTGTGTCTTTCCCGTGGACC -3′, *Il1β*-R: 5′- CAGCTCATATGGGTCCGACA -3′, *Il1ra*-F:5′- TCACCCATGGCTTCAGAGGCAGCC -3′, *Il1ra*-R: 5′- GGCCTTTCTCAGAGCGGATGAAG -3′, *Inos*-F: 5′- CCAAGCCCTCACCTACTTCC -3′, *Inos*-R: 5′- CTCTGAGGGCTGACACAAGG -3′. PCR (denaturation at 95 °C for 10 min, followed by 40 cycles of 95 °C (15 s) and 60 °C (60 s)) were performed on an ABI7300 (Applied Biosystems). Relative target gene quantification was normalized to GAPDH as housekeeping gene control for each sample and to untreated sample for each experiment, using the 2 ^–ΔΔCt^ method.

### ELISA assays

Muscles obtained at day 0, 1, 4, 10 and 21 after notexin-injury were stored at −20 °C, thawed and centrifuged to obtain tissue homogenates (1 muscle in 1 ml PBS). Murine ELISA CX3CL1 (Fractalkine) and CCL2 (MCP1) assays were performed using Quantikine ELISA Mouse Immunoassay kits (Bio-techne) following the manufacturer's protocol. ApoE3 was measured in mouse serum on day 8 post injury with a HRP-human ApoE3 ELISA Kit (Mabtech).

### Western blot

Macrophages sorted from injured muscle on day 4 post injury through MACS technology were pelleted and lysed in PBS pH7.4, 2% Triton X-100, 1% SDS complemented with antiprotease cocktail (Roche). The samples were then assayed for protein content, diluted in sample loading buffer and heated for 5 min at 95 °C. Proteins (45 μg per lane) were separated by 4-20% standard SDS–polyacrylamide gel electrophoresis. Blots were probed with polyclonal antibodies against ApoE (Abcam, ab20874, 1:750) and tubulin (Millipore, CP06, 50 ng ml^−1^).

### Immunolabelling

Frozen muscle cryosections were stained using rabbit anti mouse-MyoD (M-318, SantaCruz) and monoclonal mouse anti-myogenin (F5D, DSHB) antibodies (green) were visualized with Alexa Fluor-594- and -488-conjugated secondary antibodies (Invitrogen) before mounting in Vectashield mounting medium containing 4,6-diamidino-2-phenylindole (Vector Laboratories). Macrophages sorted from injured muscle on day 4 post injury through MACS technology were spun onto glass slides and kept at −80 °C until use. Apolipoprotein E immunolabelling used a rabbit polyclonal anti-mouse ApoE (Abcam, 1/30^e^), and an anti-rabbit Alexa 488 antibody (Life Technology) as a secondary antibody.

### BM transplantation

BM transplantation was performed as follows: donor BM cells were obtained by flushing femoral and tibial BM of WT, *Ccl2*^−/−^, *Cx3cr1*^−/−^, *Cx3cr1*^*GFP/GFP*^ or *Cx3cr1*^*GFP/GFP*^ ApoE^−/−^ mice with PBS. Cells were filtered, washed twice and counted in a Malassez slide. 10 × 10^6^ BM cells in 0.1 ml of PBS was injected retro-orbitally to 10.0-Gy-irradiated *Ccl2*^−/−^ mice (5 Gy twice, 3 h apart) directly, at the end of the second irradiation. Chimerism was evaluated on mice engrafted with *CX3CR1*^*gfp/gfp*^ or *CX3CR1*^*gfp/gfp*^ApoE^−/−^ BM, calculating the percentage of GFP^+^ cells among the total number of CD11b^+^ CD115^+^ F4/80^+^ monocytes. Muscle injury was performed 10 weeks after BM transplantation.

### Macrophage and MPC culture

Macrophages were obtained from BM precursor cells. In brief, total BM was obtained from mice by flushing femur and tibia BM with DMEM. Cells were cultured in a DMEM-containing conditioned medium of L929 cell line (enriched in M-CSF) for 7 days. After washing, DMEM-serum-free medium was added for 24 h, and cells were recovered and centrifuged in order to obtain macrophage-conditioned medium. For phagocytosis studies, cell cultures were performed under standard conditions in DMEM/F12 (Gibco) containing 20% FBS and 2% Ultroser G serum (Pall) during 16 h with or without addition of recombinant recombinant human cytokines CX3CL1 (R&D Systems) or CCL2 (PeproTech) at 100 nM.

Murine MPCs were obtained from TA muscle and cultured with standard conditions in DMEM/F12 (Gibco) containing 20% FBS and 2% Ultroser G serum (Pall). For proliferation studies, MPCs were seeded at 10,000 cells per cm^2^ on Matrigel (1/10) and incubated for 1 day with macrophage-conditioned medium+2.5% FBS. Then, cells were incubated with anti-ki67 antibodies (15580, Abcam). For fusion index studies, MPCs were seeded at 30,000 cells per cm^2^ on Matrigel (1/10) and incubated for 3 days with macrophage-conditioned medium containing 2% horse serum. Then, cells were incubated with antibodies against desmin (32362, Abcam).

### Phagocytosis

Macrophage-enriched muscular cell suspensions extracted at day 4 post injury were cultured for 4 h with red fluorescent (580/605) Fluospheres carboxylate-modified microspheres (0.2 μm size) at a 1:10 ratio (macrophage/microspheres) in six-well plates. Cells were analysed on a Zeiss inverted fluorescent axio observer Z1 microscope. Phagocytosis was expressed as the number of macrophages containing at least one microsphere per 1,000 cells, counted using and Image J software, in three distinct experiments. For some experiments, macrophage-enriched muscular cell suspensions were cultured for 16 h with or without addition of Apolipoprotein E3 (Leinco Technologies) at 10 μg ml^−1^, recombinant human cytokines CX3CL1 (R&D Systems) or CCL2 (PeproTech) at 100 nM.

*In vivo* phagocytosis was assessed on day 4 post injury as the percentage of phagocytosed myofibers (infiltrated by mononuclear cells) among the total number of necrotic (not infiltrated)+phagocytosed myofibers. In some case, Apolipoprotein E3 was injected in the muscle, twice per day (2 × 10 μg), for 3 days.

### Statistical analyses

Data are presented as the mean plus the standard error of the mean (s.e.m.). Statistical analyses compared the groups with a Mann–Whitney test or with a Wilcoxon test for matched paired values (Prism software). *P* values of less than 0.05 were considered statistically significant.

## Additional information

**Accession number**: Microarray data have been deposited in the Gene Expression Omnibus (GEO) database (http://www.ncbi.nlm.nih.gov/gds) under accession code GSE73473.

**How to cite this article:** Arnold, L. *et al*. CX3CR1 deficiency promotes muscle repair and regeneration by enhancing macrophage ApoE production. *Nat. Commun.* 6:8972 doi: 10.1038/ncomms9972 (2015).

## Supplementary Material

Supplementary Information Supplementary Figures 1-10.
