# Supplementary material for: CX3CR1 deficiency promotes muscle repair and regeneration by enhancing macrophage ApoE production
Source: Nat Commun. 2015 Dec 3;6:8972. doi: 10.1038/ncomms9972 (PMC4686853; doi:10.1038/ncomms9972)
Supplement: Supplementary Information — Supplementary Figures 1-10. [file ncomms9972-s1.pdf]

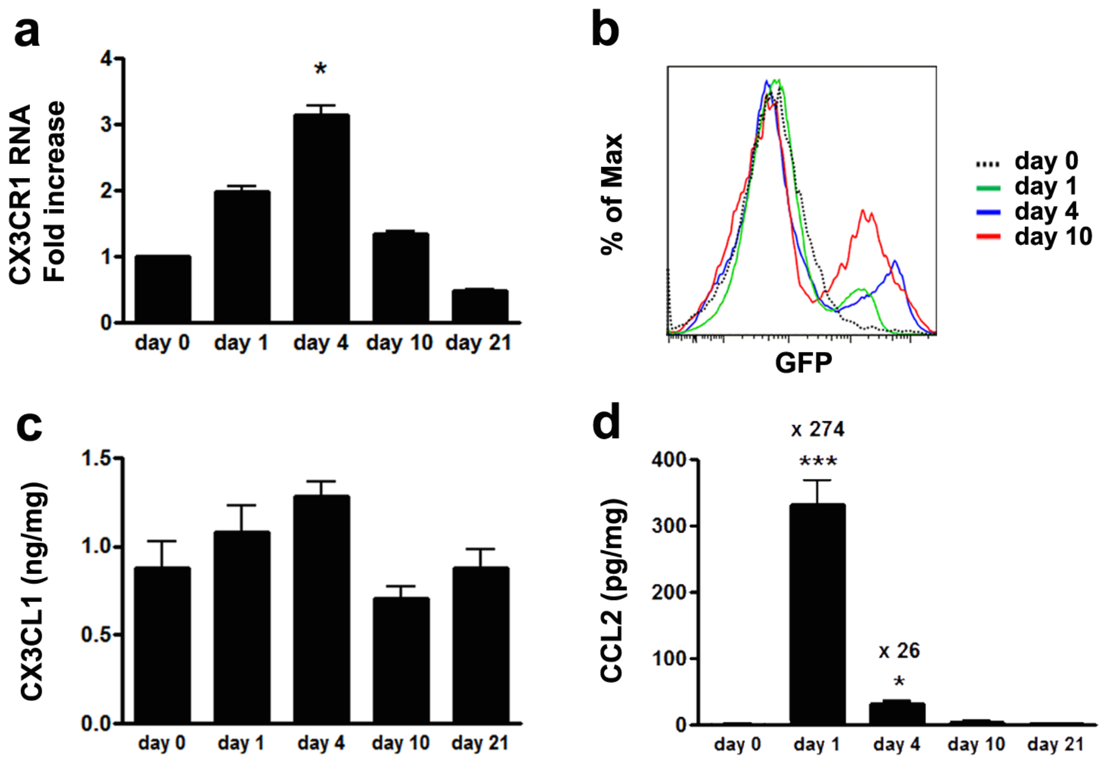

Supplementary Figure 1

Chemokine and chemokine receptor expression during muscle regeneration

(a) Analysis of CR3CR1 mRNA expression by real time-PCR at day 0, 1, 4, 10 and 21 post- muscle injury. **(b)** Mean of fluorescence intensity of GFP expression measured by flow cytometry in muscular cell suspension of *Cx3cr1*$^{gfp/+}$ mice at day 0 (black dotted line), day 1 (green), day 4 (blue) and day 10 (red) post-injury (one representative experiment out of 3) **(c)** CX3CL1 and **(d)** CCL2 proteins detection by ELISA on muscle tissue lysates extracted at days 0, 1, 4, 10 and 21 post-injury. Data are mean + s.e.m. of 3 to 4 mice per time point in 2 to 4 distinct experiments. *p<0.05 and *** p<0.001 for each time point versus day 0.

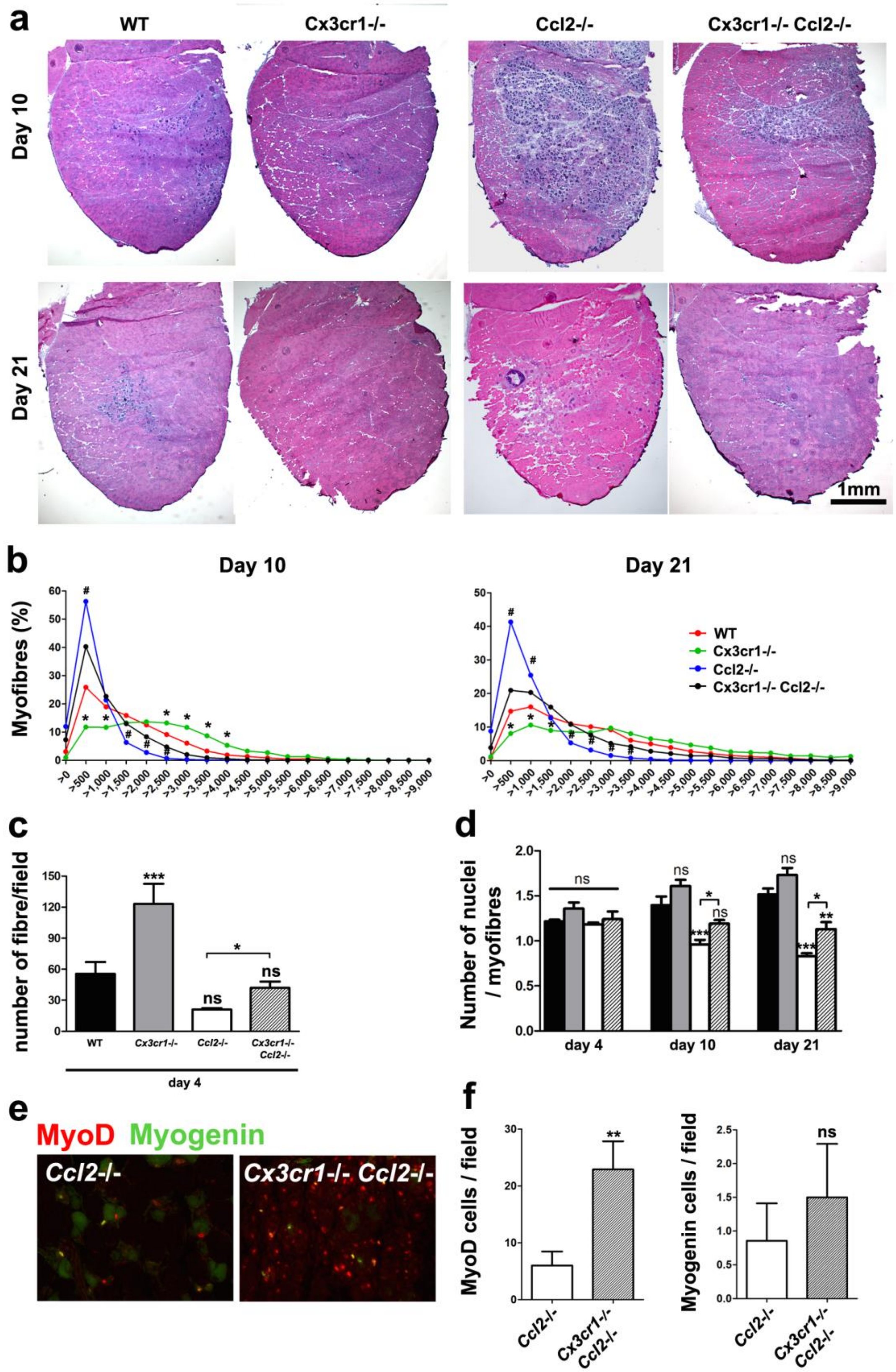

Supplementary Figure 2

Muscle regeneration in WT, *Cx3cr1*$^{-/-}$, *Ccl2*$^{-/-}$ and *Cx3cr1*$^{-/-}$ *Ccl2*$^{-/-}$ mice

(a) Representative whole muscle cross-section of injured muscle from WT, *Cx3cr1*$^{-/-}$, *Ccl2*$^{-/-}$ and *Cx3cr1*$^{-/-}$ *Ccl2*$^{-/-}$ mice on days 10 and 21 post-injury. **(b)** Distribution of CSA of regenerating myofibres

for each mouse strain on days 10 and 21 post-injury. Data are means + s.e.m. of 6 mice per strain in 2 distinct experiments. *p<0.05 for *Cx3cr1*$^{-/-}$ strain versus wild-type and $^{\#}$p<0.05 for *Ccl2*$^{-/-}$ versus *Cx3cr1*$^{-/-}$ *Ccl2*$^{-/-}$ strains. **(c)** Quantification of the total number of regenerating myofibres per field on day 4 post-injury. Data are means + s.e.m. of at least 3 different fields taken from 6 different mice per strain. **(d)** Number of myonuclei inside regenerating myofibres divided by the total number of regenerating myofibres on day 4 post-injury. Data are means + s.e.m. of at least 3 different fields taken from 6 different mice per strain. **(e)** Example of muscle cross-sections of *Ccl2*$^{-/-}$ and *Cx3cr1*$^{-/-}$ *Ccl2*$^{-/-}$ mice immunolabelled with MyoD (red) and myogenin antibodies (green) on day 4 post-injury. **(f)** Quantification of MyoD-positive and myogenin-positive cells in an average of 5 fields per mouse on day 4 post-injury (magnification of x40). Data are mean + s.e.m. of 2 different mice per strain. ns: not significant, *p<0.05, **p<0.01 and *** p<0.001 for each knock-out strain versus WT and for *Ccl2*$^{-/-}$ versus *Cx3cr1*$^{-/-}$ *Ccl2*$^{-/-}$ mice.

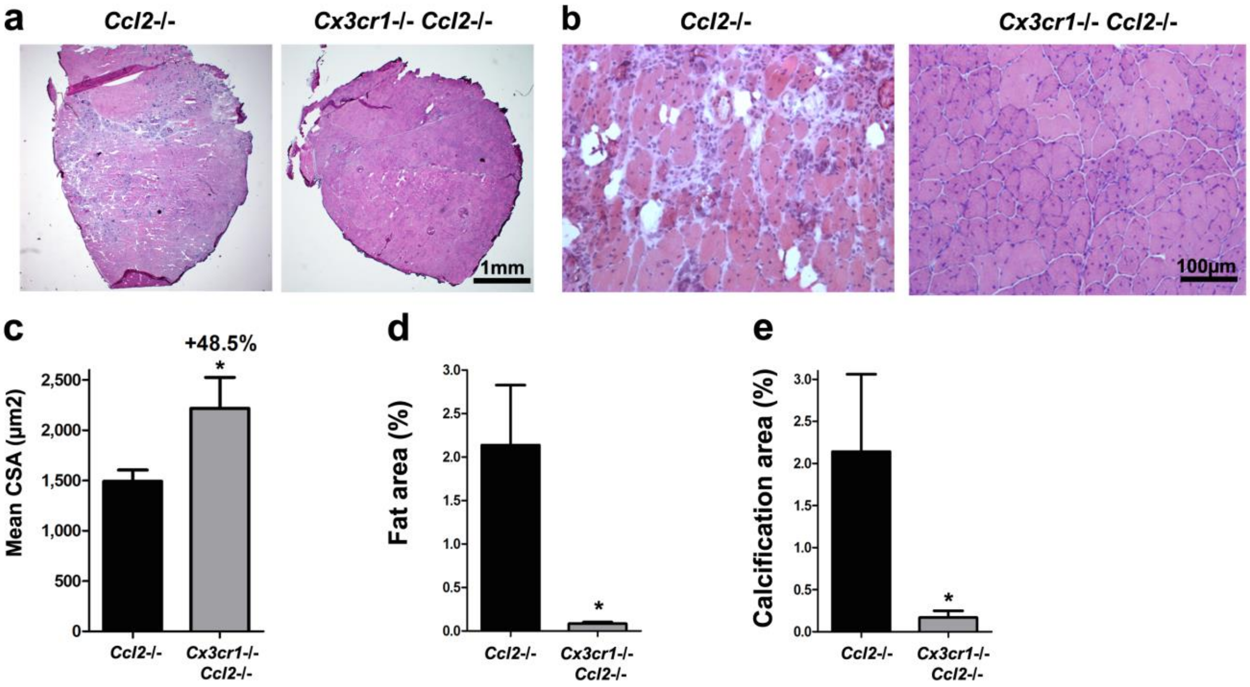

f

sex	Female		Male	
strain	*Ccl2*$^{-/-}$	*Cx3cr1*$^{-/-}$ *Ccl2*$^{-/-}$	*Ccl2*$^{-/-}$	*Cx3cr1*$^{-/-}$ *Ccl2*$^{-/-}$
Mean CSA (μm^2)	919,1	2163	1493	2217
Fat area (% of total)	3.196	1.635	2.137	0.082
Calcium deposit (% of total)	2.259	0.3422	2.14	0.168

g

Parameters (expressed as a % of Ccl2$^{-/-}$ mice)	Female	Male
Mean CSA	235%	148%
Fat area	51,2%	3,8%
Calcium deposit	15,2%	7,9%

Supplementary Figure 3

Analysis of muscle regeneration in male and female *Ccl2*$^{-/-}$ and *Cx3cr1*$^{-/-}$ *Ccl2*$^{-/-}$ mice

(a) Representative whole muscle cross-sections on day 21 post-injury of *Ccl2*$^{-/-}$ and *Cx3cr1*$^{-/-}$ *Ccl2*$^{-/-}$ male mice. **(b)** 100× magnification of representative injured muscle of *Ccl2*$^{-/-}$ and *Cx3cr1*$^{-/-}$ *Ccl2*$^{-/-}$ male mice. **(c-e)** Quantification of muscle regeneration, by measurement of the mean CSA of regenerating myofibres **(c)**, and the areas of fat **(d)** and calcification **(e)**. Results are mean + s.e.m. of 4 mice per group. **(f, g)** Comparison of muscle regeneration between *Ccl2*$^{-/-}$ and *Cx3cr1*$^{-/-}$ *Ccl2*$^{-/-}$ female/male mice on day 21 post-injury, expressed as absolute values **(f)** and percentages **(g)**.

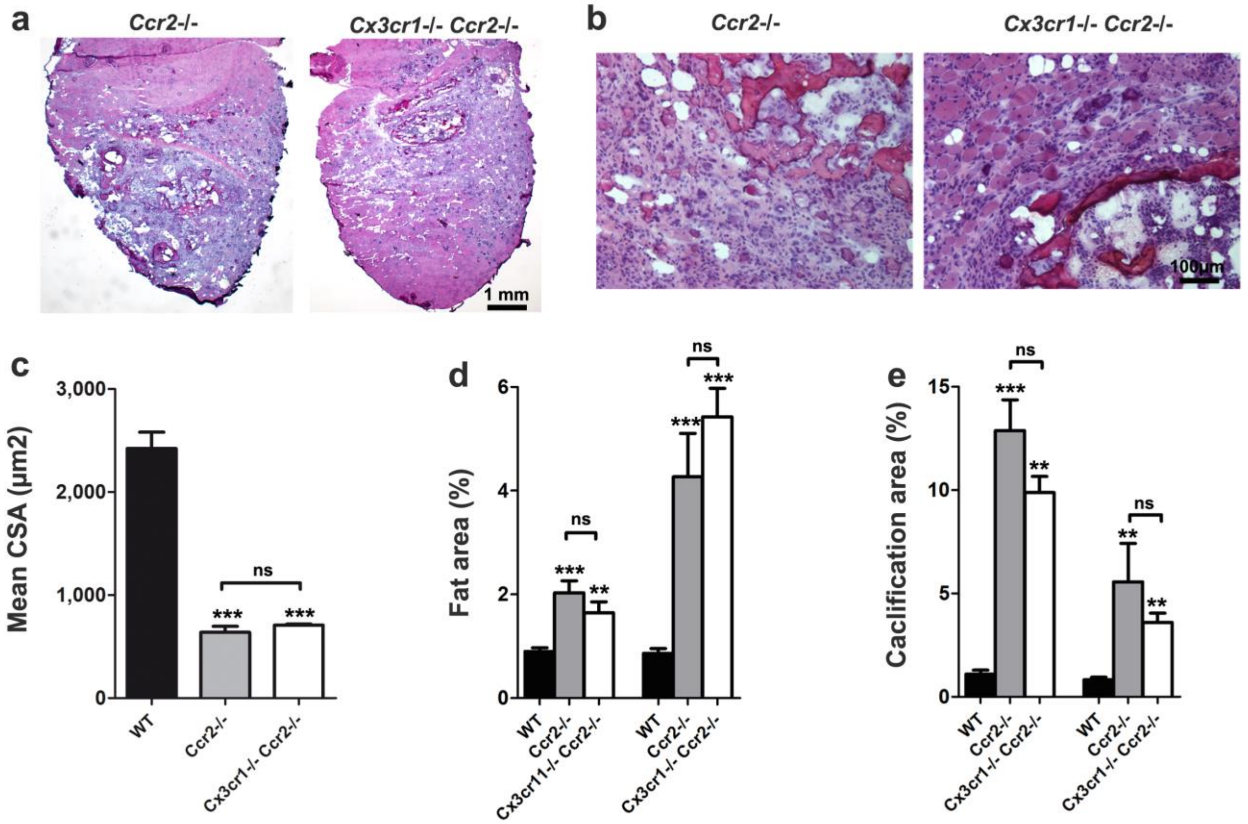

Supplementary Figure 4

Analysis of muscle regeneration in *Ccr2*$^{-/-}$ and *Cx3cr1*$^{-/-}$ *Ccr2*$^{-/-}$ mice

(a) Representative whole muscle cross-sections on day 21 post-injury of *Ccr2*$^{-/-}$ and *Cx3cr1*$^{-/-}$ *Ccr2*$^{-/-}$ female mice. **(b)** 100× magnification of representative injured muscle of *Ccr2*$^{-/-}$ and *Cx3cr1*$^{-/-}$ *Ccr2*$^{-/-}$ mice. **(c-e)** Quantification of muscle regeneration by measurement of the mean CSA of regenerating myofibres **(c)**, and areas of fat **(d)** and calcification **(e)**. Results are mean + s.e.m. of 3 mice per group. ns: not significant, *p<0.05, **p<0.01 and *** p<0.001 for each knock-out strain versus WT mice.

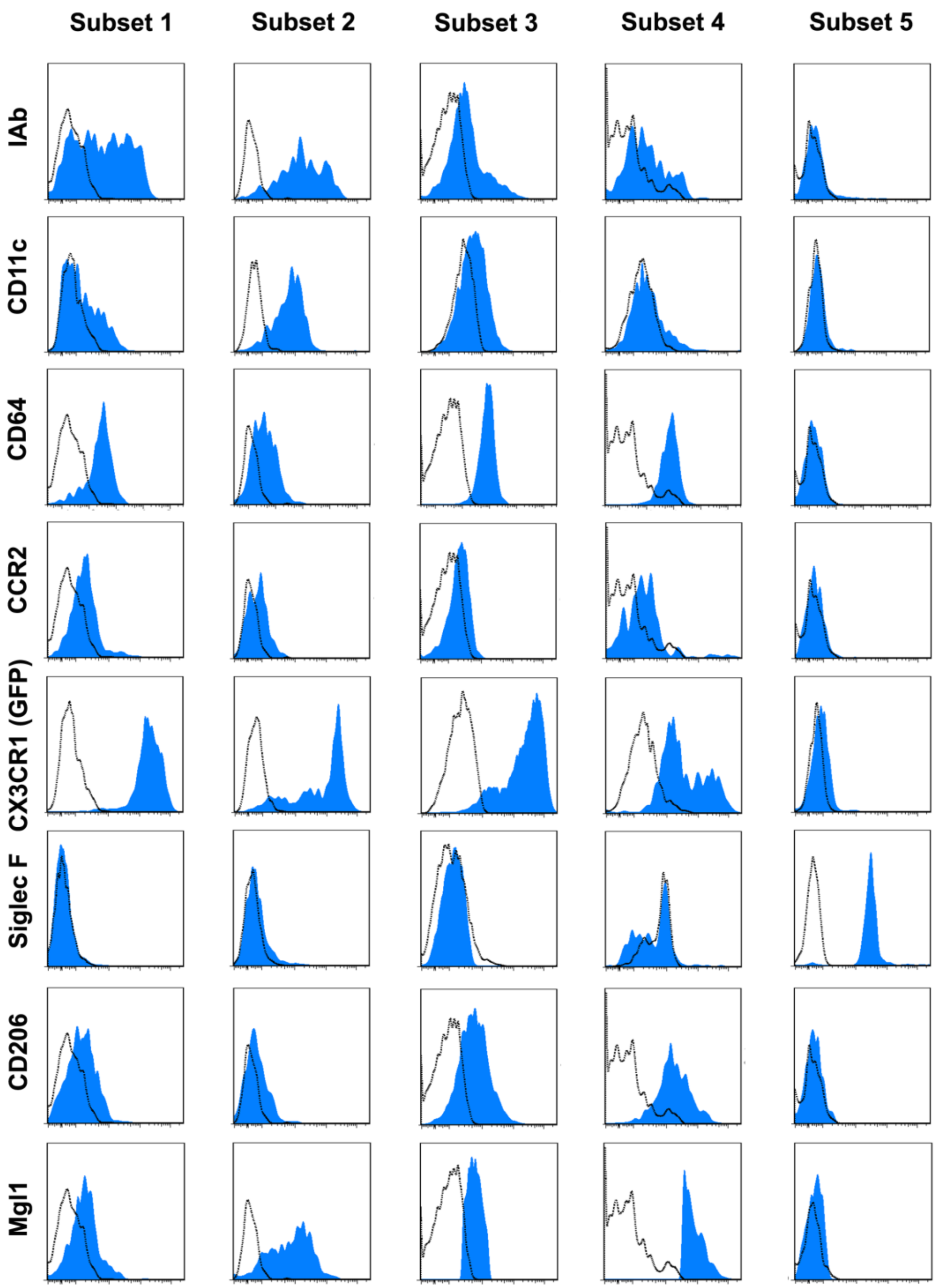

Supplementary Figure 5

Phenotypic characterization of mononuclear phagocytes in injured muscle of WT mice

Cells were been sorted from injured muscles of WT mice on day 4 post-injury after enzymatic digestion and analysed for various surface markers by flow cytometry. Subsets 1, 2, 3 and 4 of MPs identified in Figure 3 as well as F4/80$^+$ CD11b$^+$ Mgl1$^-$ cells corresponding to eosinophils were stained with haematoxylin/eosin and tested for expression of IAb (major histocompatibility complex (MHC) molecules - class II), CD11c, CD64, CCR2, CX3CR1, Siglec F, CD206 and Mgl1. Histograms of 1 representative experiment out of 3.

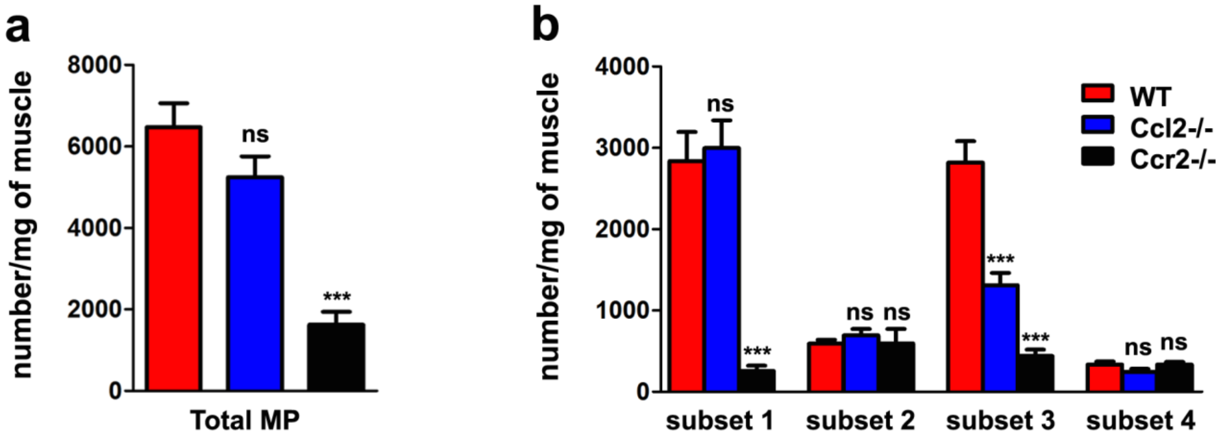

Supplementary Figure 6

Mononuclear phagocyte distribution 4 days post-injury in *Ccl2*$^{-/-}$ and *Ccr2*$^{-/-}$ mice

(a) Total number and **(b)** number per subset of MPs extracted from injured muscles of WT *(n=12)*, *Ccl2*$^{-/-}$ (n=12) and *Ccr2*$^{-/-}$ *(n=4)* mice. ns: not significant, *** $p<0.001$ for each knock-out strain versus WT mice.

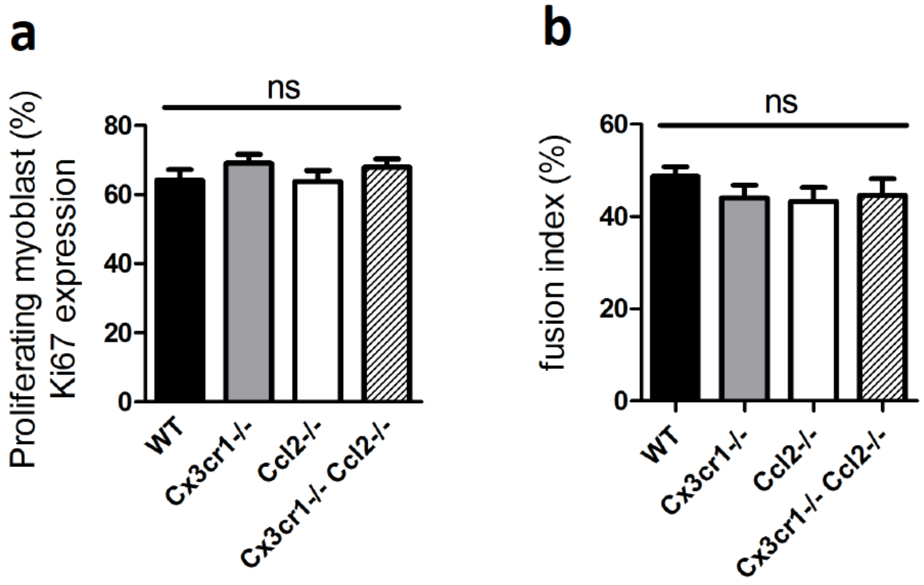

Supplementary Figure 7
Macrophages effect toward myogenesis

In vitro experiments have been performed to understand the role of CX3CR1 and CCL2 expressing macrophage toward myogenic precursor cells proliferation (Ki67 expression) and fusion (% of myoblast within myotubes vs total number of myoblast counted). **(a)** 24h conditioned media from WT, Cx3cr1$^{-/-}$, Ccl2$^{-/-}$ and Cx3cr1$^{-/-}$ Ccl2$^{-/-}$ bone marrow derived macrophages have been added on primary myogenic precursor cells and the Ki67 expression has been assessed 1 day later. **(b)** Myoblast fusion index has been assessed 4 days after macrophage conditioned media addition. Results are a mean + s.e.m. of 2 distinct experiments. ns: not significant

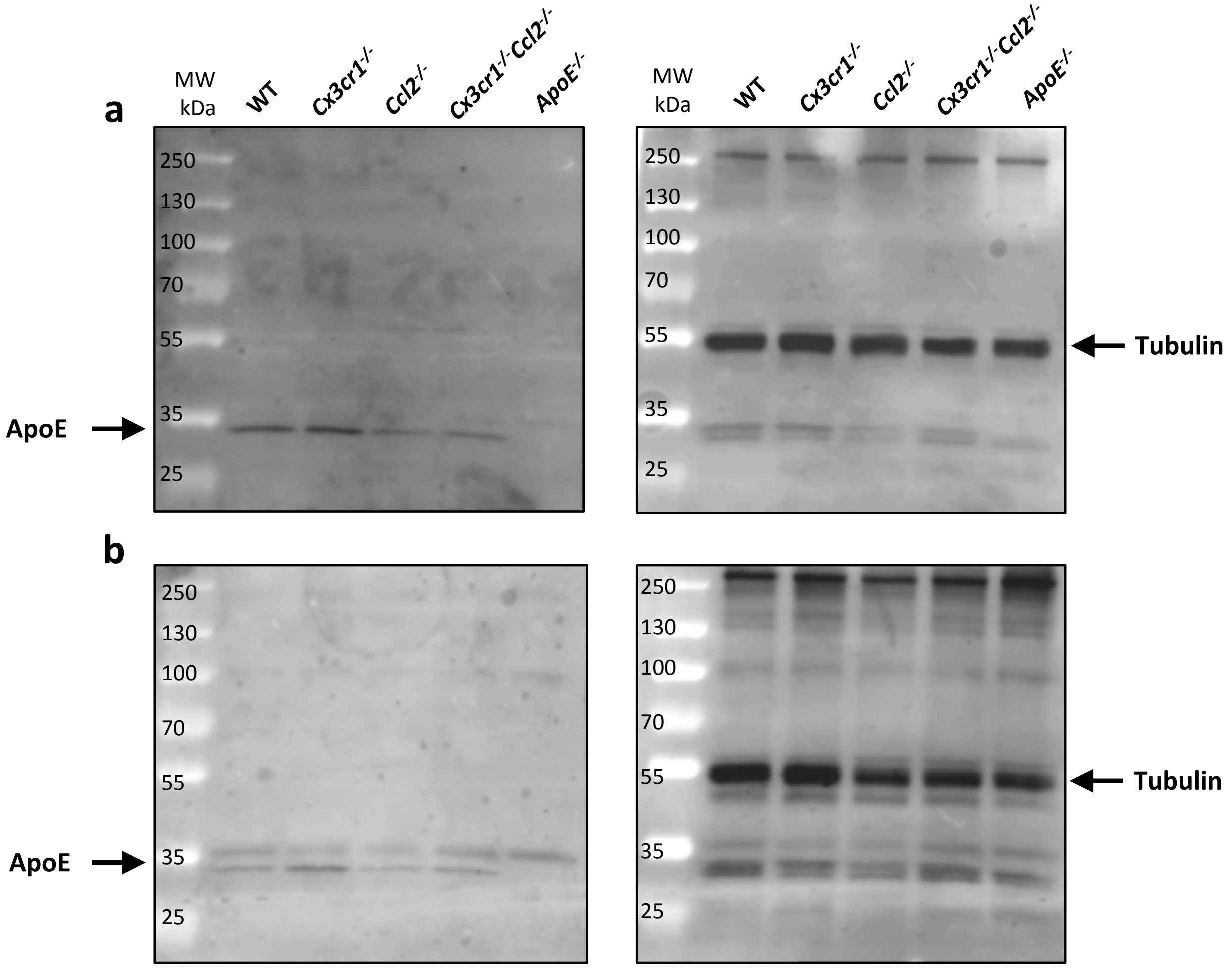

Supplementary Figure 8

ApoE expression in mononuclear phagocytes from injured muscle of WT, *Cx3cr1*$^{-/-}$, *Ccl2*$^{-/-}$ and *Cx3cr1*$^{-/-}$*Ccl2*$^{-/-}$ mice

Mononuclear phagocytes were isolated from muscle of WT, *Cx3cr1*$^{-/-}$, *Ccl2*$^{-/-}$,*Cx3cr1*$^{-/-}$*Ccl2*$^{-/-}$ and *ApoE*$^{-/-}$ mice 4 days post-notexin injection using magnetic columns (CD11b+ Ly6G- NK1.1-). Western-blots (45µg of total protein/lane) were performed using antibodies against ApoE (left panel) and against tubulin (right panel) from 2 independent experiments (a and b) (First lane: Molecular weight (MW) indicated in kDa).

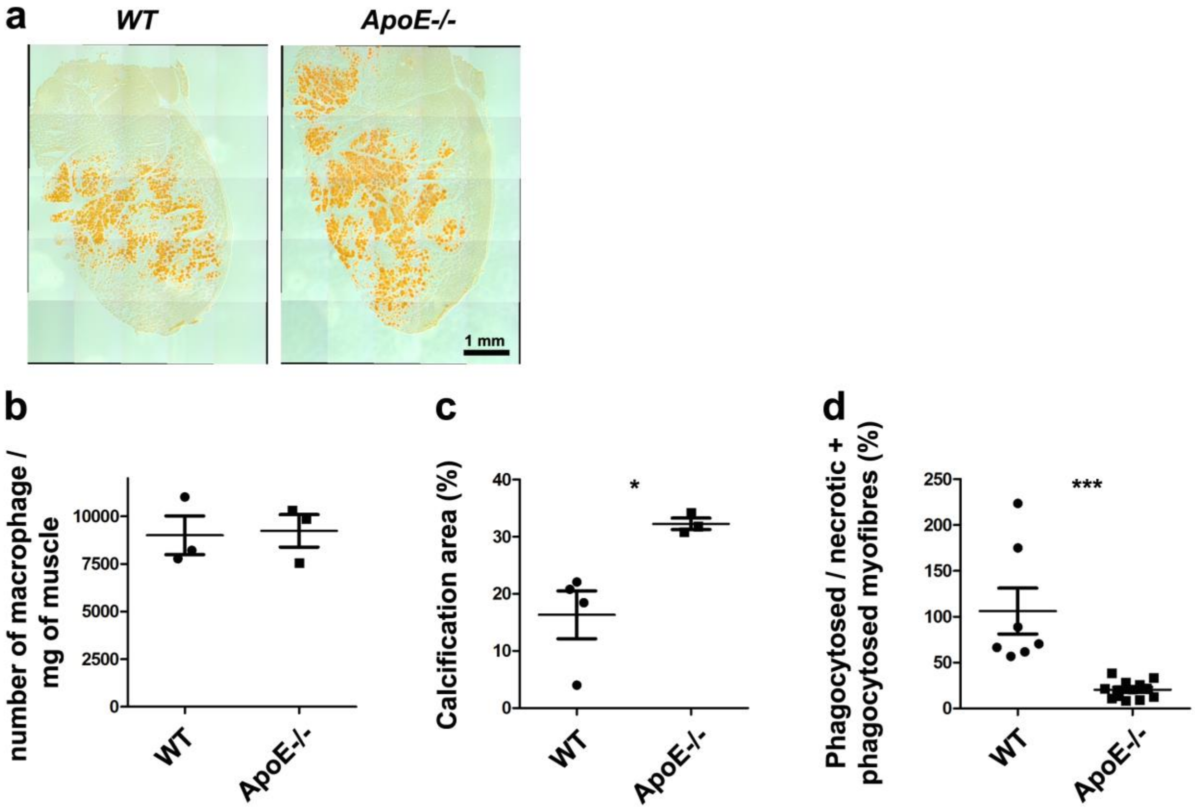

Supplementary Figure 9

Necrotic/calcified myofibres removal by mononuclear phagocytes in *ApoE$^{-/-}$* mice

(a) Representative whole muscle cross-sections on day 4 post-injury of WT and *ApoE$^{-/-}$* female mice stained with Alizarin Red. **(b)** Total number of MPs extracted from injured muscles of WT *(n=3)* and *ApoE$^{-/-}$* (n=3) mice. Quantification of muscle debris removal by assessement of the calcification area (n=3 mice per strain) **(c)** as well as the percentage of phagocytosed vs necrotic myofibres (**d)** on day 4 post-injury. WT (n=7) and *ApoE$^{-/-}$ (*n=10) mice. Results are a mean + s.e.m. of 2 distinct experiments. ns: not significant, *p<0.05 and *** p<0.001 for *ApoE$^{-/-}$* mice versus WT mice.

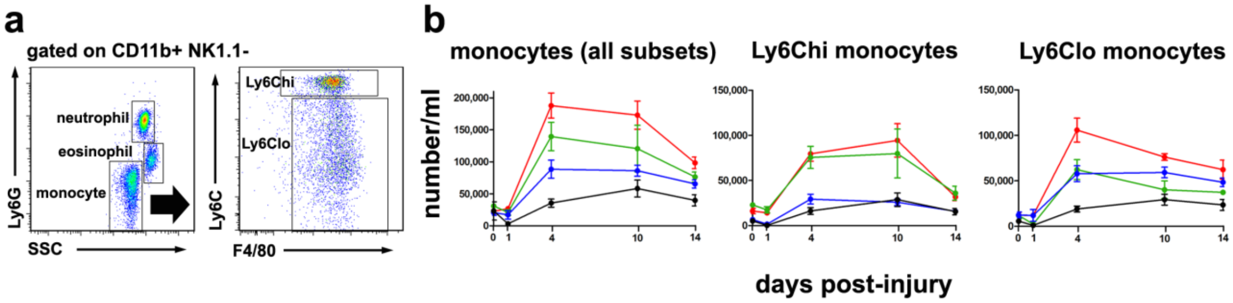

Supplementary Figure 10

Kinetics of blood monocytosis during skeletal muscle regeneration

Blood was collected in heparin, labelled with Ly6G, CD11b, F4/80, NK1.1 and Ly6C antibodies and analysed by flow cytometry. **(a)** The two subsets of blood monocytes are identified as $CD11b^{+}$ $F4/80^{+}$ $Ly6G^{-}$ $NK1.1^{-}$ SSC^{low} cells according to the expression level of Ly6C antigen. $Ly6C^{high}$ monocytes correspond to the so-called inflammatory monocytes whereas $Ly6C^{low}$ monocytes correspond to patrolling MOs. **(b)** Kinetics of blood monocytes depicted as total number and number of $Ly6C^{high}$ and $Ly6C^{low}$ monocytes during muscle regeneration in WT (red line), *Cx3cr1* (green line), Ccl2 (blue line) and Cx3cr1 Ccl2 (black line) -deficient mice. Data are mean + s.e.m. of at least 6 mice per strain and per time point in 2 to 4 distinct experiments.
